# Metabolomic Approach Based on Analytical Techniques for the Detection of Secondary Metabolites from *Humulus lupulus* L. Dried Leaves

**DOI:** 10.3390/ijms241813732

**Published:** 2023-09-06

**Authors:** Cosimo Taiti, Giacomo Di Matteo, Mattia Spano, Vittorio Vinciguerra, Elisa Masi, Luisa Mannina, Stefania Garzoli

**Affiliations:** 1 Department of Agriculture, Food, Environmental and Forest, Università di Firenze, 50019 Firenze, Italy; cosimo.taiti@unifi.it (C.T.); elisa.masi@unifi.it (E.M.); 2 Department of Chemistry and Technology of Drug, Sapienza University, 00185 Rome, Italy; giacomo.dimatteo@uniroma1.it (G.D.M.); mattia.spano@uniroma1.it (M.S.); luisa.mannina@uniroma1.it (L.M.); 3 Department for Innovation in Biological Systems, Food and Forestry, University of Tuscia, 01100 Viterbo, Italy; vincigue@unitus.it

**Keywords:** chemical composition, gas chromatography–mass spectrometry, nuclear magnetic resonance, proton-transfer-reaction time-of-flight-mass spectrometer, solid-phase microextraction, volatile organic compounds

## Abstract

Currently, the leaves of the hop plant (*Humulus lupulus* L.) are an unexploited and still little-investigated agricultural by-product. In our study, with the aim of exploring the metabolome of dried hop leaves (Chinook cultivar), a metabolomic approach was applied using multiple analytical tools such as SPME/GC–MS, GC–MS, PTR-ToF-MS, and NMR to identify the secondary metabolites. The obtained results showed the presence of a high number of components belonging to different chemical classes. In fact, thanks to the multi-methodological approach, volatile organic compounds (VOCs) with low molecular weight, terpenic compounds, fatty acids, sugars, amino acids, organic acids, and alcohols have been detected and identified. Among the revealed terpenes in the untreated matrix, the sesquiterpenes α-humulene, *β*-caryophyllene, and *α*-copaene were the most abundant. Among the saturated and unsaturated fatty acids, palmitic and linolenic acids, respectively, were those with the highest relative percentages. Particularly relevant was the sugar content, where sucrose was the main exponent while glutamate and asparagine were the principal detected amino acids. Conversely, alcohols and organic acids were the least abundant compound classes, and xanthohumol was also identified in the methanolic extract.

## 1. Introduction

The hop plant *(Humulus lupulus* L.) belonging to the Cannabaceae family is a fast-climbing perennial that grows well in temperate regions and produces large quantities of yellow–green leaves from spring to autumn.

The plant tends to climb clockwise around any support to a height of 10–18 m. The leaves are opposite or alternate and have three to five lobes with serrated margins; further, they are rough to the touch and have toothed edges.

*H. lupulus* contains several phytochemicals [1], and some of its secondary metabolites have potential value whose accumulation depends on various factors such as the genetic make-up, climatic conditions [2], and cultivation area [3]. The inflorescences represent the share of biomass exploited mainly because it is widely used to produce beer. In fact, the leaves and stems, as mass accumulated in the post-harvest period, have been little studied as they are basically considered waste material. However, with a view to a circular economy, also considering the high quantity of biomass, the recovery of this material can be useful in the industrial field as an alternative source of functional molecules [4]. In fact, previous studies have proposed the use of hop leaves as fodder for animals [5] or to produce ecological pesticides not harmful to bees [6].

Thanks to its numerous constituents, such as bitter acids, polyphenols, flavonoids, and terpenic compounds, hop cones have been extensively investigated, highlighting a wide range of biological activities including antibacterial, antioxidant, estrogenic [7,8] and, lately, also anticancer mainly linked to the presence of xanthohumol [9,10]. However, some molecules belonging to these classes of compounds have also been found in hop leaves [11,12]; among these, phenolic compounds are present in sufficient concentrations to be responsible for the antiradical capacity attributed to the leaves [13]. On the contrary, little or nothing has been reported on the volatile composition of dried hop leaves.

The Chinook hop is a cultivar well known in the American craft beer world, and to this day, the majority of the Chinook’s annual supply comes from Washington hop growers. This variety is a cross between a Petham Golding and a USDA-selected male, and it is characterized by a high content of alpha acids and good-keeping properties. It has a strong pine and resin character with spicy notes, and it can also be slightly spicy [14]. 

In this study, the complexity of chemical compounds was explored through an exhaustive analysis of the metabolome performed by combining multiple analytical tools (SPME/GC–MS, PTR-ToF-MS, and NMR). For example, the correlation between SPME/GC–MS and PTR-ToF-MS data allows a complete fingerprint of the volatolomic profile of the analyzed matrix, bypassing the limitations of each instrument. On the other hand, NMR is a powerful technique that, when applied to food, allows for the identification and quantification of secondary metabolites in a single experiment. Therefore, a metabolomic approach was applied in order to describe the volatile and non-volatile chemical profile of the dried leaves of Chinook hop grown in southern Italy.

## 2. Results

### 2.1. SPME/GC–MS Chemical Composition

Using the SPME/GC–MS technique, a total of eighteen volatile compounds, listed in Table 1, were detected and identified and the identifiers were also reported (Table S1). The volatile fraction of the dried leaves was dominated by the presence of sesquiterpenes (85.8%). Among them, *α*-humulene (27.8%), *β*-caryophyllene (19.2%), and *α*-copaene (12.9%) were the most abundant. These principal components were followed by *γ*-muurolene, *δ*-cadinene, and *β*-eudesmene, whose average percentage values ranged from 4.3 to 7.6%. The relative chromatogram is reported in Figures S1–S3.

### 2.2. PTR-ToF-MS: Determination of Volatile Compounds from Hop Dried Leaves

Hop-dried leaf samples analyzed by a Proton-Transfer-Reaction Time-of-Flight Mass Spectrometer (PTR-ToF-MS) were prepared in the same way as the ones used for Solid Phase Microextraction/Gas Chromatography–Mass Spectrometry (SPME/GC–MS) analysis with the aim to compare the results of these two methodologies. The PTR-ToF-MS data were filtered following the procedure previously described by Masi et al. [15]. Thus, all peaks imputable to water chemistry and all the interfering ions (e.g., oxygen, nitrogen monoxide) were eliminated, and subsequently, the peaks whose average concentrations were lower than 0.5 ppbv (parts per billion by volume) were also eliminated. After filtering the whole volatile organic compounds (VOCs), the spectra of the hop-dried leaf (analyzed in triplicate) were composed of 25 VOCs with different emission intensities, as reported in Table 2. All these detected compounds were identified up to *m/z* 205.195, and no other compounds with higher molecular weight were identified with an emission intensity greater than 0.5 ppbv. The revealed compounds could be tentatively attributed to compounds such as aldehydes, ketones, alcohols, terpenes, and sulfur compounds (Table 2 and the identifiers were also reported (Table S2)). The highest hop leaves volatile emissions were detected for the compounds detected at *m*/*z* 59.059 and 45.033, which showed, as a sum, over 60% of the total emission.

Some signals linked to terpene compounds were identified in the leaf samples and in accordance with what was previously reported by Stanius et al. [16]. In particular, five different signals linked to terpene emission were detected, such as *m/z* 93.069, 107.086, 109.101, 133.101, and 205.195 (Table 2). Leaves also emitted a small fraction of volatile sulfur compounds (see *m*/*z* 49.010—TI as methanethiol) probably linked to thiol compound as reported by Macchioni et al., [13]. Methanethiol is a volatile organic sulfur compound that represents one of the most frequent products of plant metabolism, as suggested by Vivaldo et al. [17].

### 2.3. Fatty Acids Content

GC–MS analyses of the dried leaves extract highlighted the presence of five fatty acids, including two saturated and three unsaturated (Table 3 and the identifiers were also reported (Table S3)). Linolenic acid (48.3%) and palmitic acid (38.7%) were the major components, followed by linoleic (5.9%), stearic (4.3%), and palmitoleic acid (2.9%). The relative chromatogram is reported in Figure S2.

### 2.4. Chemical Composition of Methanolic Extract after Derivatization

The GC analyses carried out on the silylated methanolic extract allowed the detection of twenty-five components belonging to different chemical classes (Table 4 and the identifiers were also reported (Table S4)). The sugar fraction was the most abundant since sucrose reached the highest percentage value (62.3%). Among detected alcohols, D-pinitol (2.8%) was the major component, followed by glycerol (1.1%). Detected organic acids represented a minor share, where only pyruvic acid reached 0.1%. The relative chromatogram is reported in Figure S3.

### 2.5. NMR Analysis of Dried-Hop Leaves

**Figure 1:**
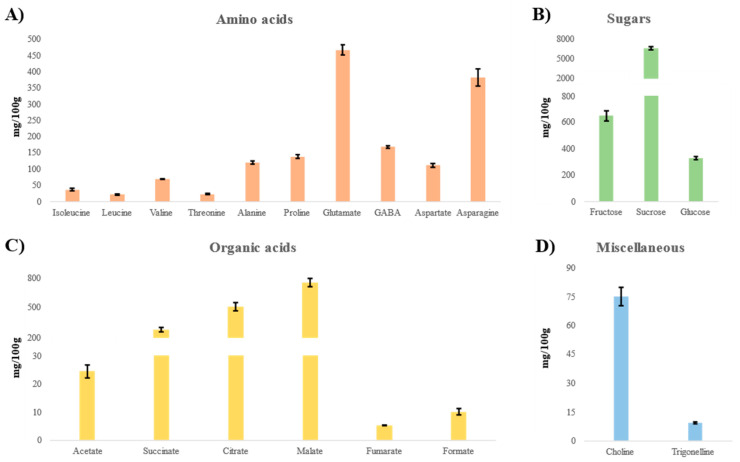
Bar charts of the metabolites identified and quantified (mg/100 g of DW ± SD) in the ^1^H NMR spectra of hydroalcoholic extracts of hop leaves. (**A**) amino acids, (**B**) sugars, (**C**) organic acids, (**D**) miscellaneous metabolites.

The complete NMR assignment of the hydroalcoholic extract of hop leaves is reported in Table 5 (the identifiers were also reported in Table S5) and Figures S4–S7, and the bar charts of the quantitative results in Figure 1. Eleven amino acids (isoleucine, leucine, valine, threonine, alanine, proline, glutamate, GABA, aspartate, asparagine, and glutamine), six organic acids (acetate, succinate, citrate, malate, fumarate, and formate), three sugars (fructose, sucrose, and glucose), and choline and trigonelline were identified. 

Among amino acids, glutamate and asparagine were the most abundant, representing 30.3% *w/w* and 24.8 % *w/w,* respectively, of the total detected amino acids. The branched-chain amino acids were the least representative (under 5% *w/w* each). GABA, aspartate, and proline completed the identified compounds of this family, of which glutamine is not quantifiable. 

Sugars represented the largest fraction of water-soluble compounds in hydroalcoholic extracts from hop leaves, and sucrose was the major one (87.2 % *w/w* of the total sugars). Many organic acids were also found, with malic, citric, and succinic acid as the main ones (47.5, 32.4, and 17.6% *w/w* of the total organic acids, respectively).

Regarding the methanolic extract, the HSQC NMR experiment was chosen to see correlations between hydrogen atoms and the carbon atom to which they are attached. In fact, by means of HSQC, it is easy to assign cross peaks common to all humulones and lupulones molecules (due to methyl and methylene proton resonances of the C-4 prenyl side chain) and cross peaks typical of each series of bitter acid (due to proton resonances methyl and methylene of the prenyl side chains in C-6) [18]. Furthermore, by HSQC, it is also easy to assign unique cross peaks for adhumulone and adlupulone [19]. No bitter acids were detected since no signals of the aliphatic portion of the humulone and lupulone bitter acids series were detected in the characteristic region between 0.89 and 1.3 ppm. However, the NMR assignment of the most abundant prenylated flavonoid xanthohumol, was reported in Table 6 and Figure S8. 

The obtained quantitative amount of xanthohumol was 5.28 g/100 g, which represents 0.00528% of the dry weight in the analyzed hop leaves. This was in agreement with what was previously reported [16], where the xanthohumol content in leaves varied with the type of cultivar and, in any case, was lower than its content in hop cones.

The obtained quantitative amount of xanthohumol was 5.28 g/100 g, representing 0.00528% of dry weight in the analyzed hop leaves. This was in agreement with what was previously reported [16], where the xanthohumol content of the leaves varied with the type of cultivar, and, in any case, it was lower than its content in the hop cones.

## 3. Discussion

In the present work, for the first time, a multi-methodological approach was applied to study the metabolome of dried Chinook hop leaves. The obtained results highlighted the presence of a high number of volatile and semi-volatile metabolites.

Overall, up to now, a small number of previous studies have focused on the chemical characterization of hop leaves, with a very small fraction of these involving the volatile content of untreated plant material. In fact, most of the previous articles mainly concerned the determination of α- and *β*-acids of hop inflorescences [20,21,22,23,24]. Furthermore, to the best of our knowledge, this is the first work reporting the fatty acid content of hop leaves beyond the investigated cultivar.

Our data showed that α-humulene followed by *β*-caryophyllene and α-copaene were the most representative terpene compounds. In our previous study [25], carried out on fresh leaves, we found that *β*-caryophyllene, α-humulene, and *δ*-cadinene, in descending order, were the most abundant. This suggests that drying, in general, may influence the volatile profile of this matrix. In addition, as reported by Macchioni et al. [13], the drying method can induce variations in the content of flavones, thiols, phenols, and pigments in hop leaves. Likewise, genetic and environmental factors greatly affect the content of phytochemical compounds [26].

The detected emission of several terpene compounds from the leaves was in line with what was previously reported by Stanius et al. [16], where borneol, *γ*-cadinene, estragole, *α*-copaene, *β*-farnesene, *β*-caryophyllene, *γ*-muurolene, and *α*-humulene were emitted by leaf samples of five Lithuanian hop varieties. These compounds were characteristic of the volatile composition of both leaf and cone hops, albeit with different trends [16].

Thanks to PTR-ToF-MS analyses, in addition to some terpenes, which represent about 2% of the total VOC emission, a range of low-molecular-weight compounds such as aldehydes, ketones, alcohols, and sulfur compounds was also found. Among them, aldehydes such as acetaldehyde (C_2_H_5_O^+^) and propanal (C_3_H_7_O^+^) were the most abundant identified compounds, whereas alcohols, ketones, and sulfur showed the lowest emission. For example, the emission amount of CH_5_S^+^ (methanethiol) accounted for 0.2% of the total VOC emission (Table 2). 

The data obtained by the GC–MS and NMR analyses on the derivatized methanolic extract and the hydroalcoholic extract, respectively, were aligned as both techniques highlighted the presence of compounds belonging to other chemical classes, such as sugars which made up the main fraction and the organic acids. Moreover, taken individually, the GC–MS technique has allowed the identification of a series of alcohols, albeit with a low relative percentage; on the other hand, the NMR technique has made it possible to identify a large number of amino acids. 

In general, lH-NMR was widely used to characterize bitter acids and xanthohumol in hop cones [18,27,28], but no NMR study is available on hop leaves. On the other hand, only one study reports the polar metabolite content in hop cones by NMR. [29]. Asparagine, glucose, and fructose have been reported to be the major amino acids and sugars in hop cones [18]. Instead, in our study, glutamate, asparagine, and sucrose were the main ones in hop leaves. Regarding bitter acids, they were not detected in hop leaves due to their low concentration [30,31]. 

The obtained results from our investigation highlighted that hop leaves can be a natural alternative source of a large number of bioactive metabolites whose biological effects are well known. In fact, the identified different phytochemical classes play a key role in the prevention of various diseases. Among the most representative compounds, the major detected sesquiterpenes *β*-caryophyllene and *α*-humulene can exert antibacterial, anti-inflammatory, antioxidant, and anticancer actions [32,33]. The fatty acids revealed in hop leaves are also known for their significant effects on human health [34]. For example, linolenic acid, as the major detected fatty acid, has important physiological actions for plants; moreover, following metabolic conversions into bioactive derivatives, it is expected that it can also treat many diseases [35]. Conversely, palmitic acid has been reported to exhibit significant antibacterial activity against oral microorganisms [36], in addition to having metabolic implications [37]. Furthermore, the structures of many well-known antibacterial and antifungal agents exhibit an amino acid backbone that is crucial for carrying out antimicrobial activity [38]. In plants, sugars are essential as, in addition to providing carbon skeletons as substrates for tissue growth, they also act as signal molecules, thus influencing metabolic processes [39]. Among the detected alcohols, D-pinitol, a glucose metabolite widely identified in plants of the Leguminosae family, has been reported to possess several pharmacological activities, including antidiabetic, antioxidant, anti-inflammatory, and chemo-preventive effects [40]. Finally, xanthohumol, the identified polyphenol in hop leaves, exerts various pharmacological effects such as anti-inflammatory, antioxidant, hypoglycemic, and even anticancer activity [41].

## 4. Materials and Methods

### 4.1. Plant Material

Dried hop leaves from the Chinook cultivar were grown in San Nicandro Garganico (Foggia, Italy) at the Vocino family farm in 2021. 

The drying of hop leaves took place in a natural way, inside a dark and ventilated room with a constant humidity level (about 50% RH). After about 3 days, the leaves were vacuum-packed and stored in a cool and dry place. Before the analysis, the dried hop leaves were finely chopped with a grinder (MulinexAR 11, Groupe SEB, France) with a particle size of approximately 1.0 mm. 

### 4.2. SPME Sampling

Using the SPME sampling technique, the volatile profile of the untreated matrix was described. About 2 g of the dried leaves were placed inside a 15 mL glass vial with a PTFE-coated silicone septum. A SPME device from Supelco (Bellefonte, PA, USA) with 1 cm fiber coated with 50/30 μm DVB/CAR/PDMS (divinylbenzene/carboxen/polydimethylsiloxane) was chosen to extract the components. The operative conditions followed Nezi et al. [42] with minor modifications. Component desorption was ensured by inserting the fiber directly into the GC injector maintained at 250 °C in splitless mode.

### 4.3. GC–MS Analysis of the Dried Hop Leaves

To carry out the analyses of the headspace hop leaves, a Clarus 500 model Perkin Elmer (Waltham, MA, USA) gas chromatograph coupled with a mass spectrometer equipped with a FID (flame detector ionization) was used [25,43]. A Varian (VF-1 ms) apolar column was used, and the detected and identified components are listed in Table 1.

The oven programmed temperature was set initially at 55 °C and then increased to 220 °C at 6°/min and finally held for 15 min. Helium was used as carrier gas at a constant rate of 1 mL/min. MS detection was performed with electron ionization (EI) at 70 eV, operating in the full-scan acquisition mode in the *m*/*z* range of 35–550 amu. To identify the volatile compounds, the MS-fragmentation pattern obtained was compared with those of pure components stored in the Nist 02 mass spectra library database. The Linear Retention Indices (LRIs) were also calculated using a series of alkane standards (C_8_–C_30_ n-alkanes, Supelco, Bellefonte, CA, USA) and then compared with those available in the literature. Semi-quantitative values (peak area percentages) were obtained by peak normalization without using correction factors. The area of each peak in the chromatographic profile was divided by the total sum of all peaks within the chromatogram. The normalized data was then multiplied by 100 and expressed as a percentage. Analyses were carried out in triplicate.

### 4.4. Extraction and Transesterification Processes

A 3.0 g sample of the dried and chopped hop leaves was weighed in a flask and extracted with 10.0 mL × 2 of methanol (Carlo Erba, Milan, Italy) at room temperature. After separation from solid residue by filtration, the methanolic solutions were combined and dried under reduced pressure at 30 °C to obtain 0.301 g of solid extract.

A 20 mg extract was dissolved in toluene (4 mL) and transferred into a tube to carry out the transesterification [44]. In detail, a solution (10 mL) of 1% (*v*/*v*) concentrated sulfuric acid in methanol was added, and the stoppered tube was left overnight at 50 °C. The solution was cooled and transferred in a separatory funnel, and 20.0 mL of water with 5% NaCl was added. A 20.0 mL × 2 of n-hexane (Carlo Erba, Milan) was used to extract the esters, the upper solutions were combined, washed with aqueous potassium bicarbonate (2%) (Merck, Darmstadt), filtered over anhydrous sodium sulfate (Merck, Darmstadt, Germany) and dried under reduced pressure at 30 °C.

### 4.5. GC–MS Determination of Fatty Acids (FAs) Content

To describe the FAs content, the same apparatus reported above (Section 4.3) was used. The GC operative conditions to analyze the transesterified extract were as follows: the injector was set to 280 °C, and the oven temperature was programmed from 170 °C at a rate of 3 °C/min to 260 °C for 15 min. 2 µL of the extract was injected into the column in splitless mode. The identification and quantification of the components were performed as previously described. Analyses were carried out in triplicate.

### 4.6. GC–MS Analysis of Methanolic Extract (after Derivatization)

To describe the chemical content of methanolic extract, approximately 1 mg was added of 300 µL of pyridine and 100 µL of bis-(trimethylsilyl) trifluoroacetamide (BSTFA) with heating at 80 °C for 50 min. 1 μL of the silylated sample was manually injected at 280 °C into the GC injector in splitless mode [45]. The analysis was performed using the same apparatus, GC-FID/GC–MS. The oven temperature program was as follows: 70 °C then a gradient of 6 °C/min to 170 °C, a gradient of 7 °C/min to 250 °C, and a gradient of 8 °C/min to 300 for 15 min. The compound identification was based on the percentage of similarity plus comparison of mass spectra (MS) using the software NIST02 (Gaithersburg, MD, USA) data library. Relative percentages for quantification of the components were calculated by electronic integration of the GC-FID peak areas with respect to the percent total of ion chromatograms (TIC%). Analyses were carried out in duplicate.

### 4.7. PTR-ToF-MS Analysis of Dried Leaves

The volatile profile of the dried hop leaves was performed using, for the first time, a PTR TOF 8000 model (Ionicon Analytik GmbH Innsbruck, Innsbruck, Austria). The ion H_3_O^+^ was used as a reagent ion for the proton transfer reaction. The use of a PTR-TOF-MS allows the simultaneous detection of a wide range of VOC with a high mass power [46]. The sample preparation and experimental set-up procedure was followed as previously conducted by Masi et al. [15] on *Mentha spicata* dried leaves. 

Subsequently, the weighed samples (five grams of chopped dried leaves) were transferred into a glass jar (1/3 L) and incubated for 60 s. The headspace was sampled at a fixed flow rate of 50 sccm for 3 min. Five replicates of dried leaf hops were analyzed, and between the measurements, the PTR inlet line was flushed with clean air to avoid the memory effect. The experiment was led using the following drift tube parameters: voltage 594 V, temperature 110 °C, extraction voltage at the end of the drift tube (Udx) 35 V, and the acquisition was set to 1 mass spectrum per second. Mass spectral data were collected each second over a mass range between 20 and 220 *m*/*z* using a ratio E/N = 140. In order to have high mass accuracy and easily determine the chemical formula of each peak detected, the internal calibration was performed off-line, using together with known low mass ions (NO^+^ peak *m*/*z* = 29.997 and C_3_H_7_O^+^ peak *m*/*z* = 59.049) a known compound with high mass ions as the carvone (C_10_H_15_O^+^ peak *m*/*z*= 151.075). Then, once the chemical formula was identified, each signal detected was tentatively assigned to a specific compound based on the results previously obtained by the same tool or by GC–MS analysis and of the available literature on the volatile compounds emitted from hop leaf products. Lastly, raw data acquisition and processing were conducted using TofDaq software (20th Tofwerk AG, Thun, Switzerland) and the methodology used is reported by Taiti et al. [47].

### 4.8. NMR Analysis

For the untargeted NMR analysis, two types of extraction protocols were performed on the dried hop leaves: the Bligh–Dyer extraction and a methanol extraction.

The first one is a useful extraction method for the isolation of most polar metabolites, obtaining a clean extract free from traces of organic compounds. This extraction was performed according to previous literature articles [48,49,50]. In brief, 0.100 g of the dried hop leaves were sequentially added with 0.8 mL of Millipore grade water and 3 mL of methanol/chloroform (2:1% *v*/*v*). The extraction was performed through a sonication step at room temperature for 10 min. Then, the extract was sequentially added with 1 mL of chloroform and 1 mL of Millipore grade water to obtain the biphasic system methanol/chloroform/water (2:2:1.8% *v*/*v*/*v*). Finally, the upper hydroalcoholic phase was collected, and the residue pellet was extracted again. The obtained extract was dried under N_2_ flow and prepared for the NMR analysis. The dried hydroalcoholic phases were solubilized in a solution of 20 mM phosphate buffer/D_2_O containing 0.4 mM 3-(trimethylsilyl)-propionic-2,2,3,3-d4 acid sodium salt (TSPA) and EDTA-d16, and 0.7 mL was transferred in a 5 mm NMR tube.

Regarding the methanolic extraction, the used protocol is an already established method for hops bitter acids and xanthohumol extraction [30]. In detail, 0.100 g of dried leaves were extracted with 1 mL of MeOD with 10 min of sonication at room temperature. The TSPA internal standard was added to achieve a 1 mM concentration in each extract and directly analyzed by NMR. 

Both the extraction methods were performed in triplicate and analyzed with a JNM-ECZ 600R (JEOL Ltd., Tokyo, Japan) spectrometer operating at the proton frequency of 600.17 MHz equipped with an autosampler.

The parameters used for the hydroalcoholic fraction analysis were the following: 90° pulse angle of 9.26 μs, 128 transients, 4 pre-scans, 65 K data points, and a presaturation pulse sequence to suppress the water signal. Regarding the methanol extract, a double suppression at 4.83 ppm and 3.28 ppm was performed. The other experimental parameters were a 90° pulse angle of 9.0 μs, 128 transients, 4 pre-scans, and 65 K data points. 

2D NMR experiments, namely ^1^H-^1^H TOCSY and ^1^H-^13^C HMBC for the hydroalcoholic extract and ^1^H-^13^C HSQC for the methanolic one were performed using previously reported experimental parameters [51]. All the NMR spectra were processed using the JEOL Delta v5.3.1. software (JEOL Ltd., Tokyo, Japan). The 1H NMR spectra were Fourier transformed, manually phased, and automatically base corrected. Both types of spectra were referenced to the internal standard TSPA set at 0.00 ppm.

The metabolites identification was based on literature NMR data on other plant matrices and hop parts [18,52,53], 2D experiments, and standard additions. Signals of the identified metabolites and the internal standard in the hydroalcoholic and methanolic extracts were manually integrated for all the samples. In order to realize an absolute quantification, the area of each signal was referred to as the area of the TSP methyl group, the internal standard with a known concentration of 0.4 mM, taking into account the number of protons (Supplementary Material). The final quantitative results were expressed as mg/100 g of DW ± SD.

### 4.9. Statistical Analysis

Statistical analyses were performed with GraphPad Prism 6.1 software. The experiments were replicated three times unless otherwise indicated, and the data were expressed as the average ± standard deviation (SD). 

## 5. Conclusions

Until recently, hop leaves, considered waste material, were less scientifically considered than the cones or the female inflorescences. In our study, with the aim of obtaining compositional information on the dried leaves of the Chinook cultivar, a metabolomic approach was carried out. The findings showed a considerable and diversified content of secondary metabolites, many of which are already known for their bioactive potential.

A deeper knowledge of the metabolome, in the context of a circular economy, is useful for understanding how to better exploit this matrix that has so far been underused in various sectors such as industrial, agricultural, or food. Therefore, this information is fundamental to hypothesize the use of the hop leaves as raw material and for contributing to the enhancement of the cultivation of the hop plants thanks to the multiplicity of uses of its products. In conclusion, this study highlights the usefulness of combining different analytical techniques to fully investigate the metabolomic profile of any useful part of the plant.

## Figures and Tables

**Table 1:** Chemical volatile composition (percentage mean value ± standard deviation) of the dried hop leaves as determined by SPME/GC–MS.

N°	COMPONENT ^1^	LRI ^2^	LRI ^3^	Chinook (%)
1	isobutyric acid	762	765	1.4 ± 0.04
2	propionic acid	968	972	0.7 ± 0.02
3	5-hepten-2-one, 6-methyl-	981	986	1.9 ± 0.03
4	2-methylbutyl isobutyrate	986	989	8.6 ± 0.03
5	*β*-myrcene	988	991	0.4 ± 0.02
6	amyl isovalerate	1090	1093	0.8 ± 0.02
7	*β*-cyclocitral	1192	1197	0.4 ± 0.01
8	ylangene	1368	1376	3.4 ± 0.02
9	*α*-copaene	1385	1392	12.9 ± 0.05
10	*β*-bourbonene	1392	1390	0.5 ± 0.01
11	*β*-caryophyllene	1438	1440	19.2 ± 0.09
12	*α*-humulene	1475	1473	27.8 ± 0.11
14	*γ*-muurolene	1480	1486	7.6 ± 0.02
13	*β*-eudesmene	1490	1488	4.3 ± 0.03
15	*α*-selinene	1495	1493	1.8 ± 0.02
16	*γ*-cadinene	1510	1509	2.2 ± 0.02
17	*δ*-cadinene	1513	1505 ^§^	5.7 ± 0.03
18	selina-3,7(11)-diene	1535	1530	0.4 ± 0.01
	SUM			100.0
	Monoterpenes			0.4
	Diterpenes			0.4
	Sesquiterpenes			85.8
	Others			13.4

^1^ The components are reported according to their elution order on apolar column (VF-1ms); ^2^ Linear Retention Indices measured on apolar column; ^3^ Linear Retention Indices from literature; ^§^ Normal alkane RI.

**Table 2:** Chemical composition (mass charge, chemical formula, tentative identification, average emission, standard deviation, percentage emission for each *m/z* detected) of *H. lupulus* dried leaves identified via PTR-ToF-MS. Data were expressed as average of three replicates (±SD) and as % of the total.

N° of Compounds	*m*/*z*	Chemical Formula	Tentative Identification	Average Samples Emission	Standard Deviation (SD)	Emission (%)
1	27.022	C_2_H_3_^+^	Acetylene	21.82	2.60	3.03
2	31.018	CH_3_O^+^	Formaldehyde	50.21	4.64	6.96
3	33.033	CH_5_O^+^	Methanol	22.79	3.95	3.16
4	41.038	C_3_H_5_^+^	Alkylic fragment	11.69	2.16	1.62
5	43.018	C_2_H_3_O^+^	Aldehyde fragment	31.53	10.18	4.37
6	43.054	C_3_H_7_^+^	General alkane/VOC fragment	26.83	4.17	3.72
7	45.033	C_2_H_5_O^+^	Acetaldehyde	229.17	61.98	31.79
8	47.013	CH_3_O_2_^+^	Formic acid/formates	9.19	1.34	1.27
9	49.011	CH_5_S^+^	S Compound (methanethiol)	1.45	0.21	0.20
10	55.054	C_4_H_7_^+^	Fragment	7.12	1.44	0.99
11	57.069	C_4_H_9_^+^	Alcohol fragment	3.58	0.78	0.50
12	59.049	C_3_H_7_O^+^	Propanal, Acetone	236.97	52.32	32.87
13	61.028	C_2_H_5_O_2_^+^	Acetates	21.72	3.61	3.01
14	69.069	C_5_H_9_^+^	Isoprene/Cycloalkane fragment	5.87	1.39	0.81
15	71.049	C_4_H_7_O^+^	Butenal	1.7	0.41	0.24
16	73.065	C_4_H_9_O^+^	Isobutanal/butanone/methylpropanal	13.76	4.23	1.91
17	83.086	C_6_H_11_^+^	C6 compounds (hexenal, hexenols)	1.16	0.57	0.16
18	85.065	C_5_H_9_O^+^	Methyl butenal	2.27	0.53	0.31
19	87.044	C_4_H_7_O_2_^+^	2,3-Butanedione; Butyrolactone	1.06	0.31	0.15
20	87.080	C_5_H_11_O^+^	Pentanal/3-methylbutanal	11.87	3.28	1.65
21	93.069	C_7_H_9_^+^	Terpene fragment	2.28	0.37	0.32
22	107.086	C_8_H_11_^+^	Terpene fragment	1.69	1.06	0.23
23	109.101	C_8_H_13_^+^	Terpene fragment	2.10	0.55	0.29
24	133.101	C_10_H_13_^+^	Terpene fragment	1.5	0.34	0.21
25	205.195	C_15_H_25_^+^	Sesquiterpenes like compounds	1.58	0.40	0.22
Total Terpene Emission (ppbv)				9.14	2.19	1.26
Total VOCs Emission (ppbv)				720.90	152.55	100.00

**Table 3:** FAs content (percentage mean value ± standard deviation) of the transesterified extract, as determined by GC–MS.

N°	COMPONENT ^1^	LRI ^2^	LRI ^3^	(%)
1	palmitoleic acid, C16:1*n*7		1930	2.9 ± 0.02
2	palmitic acid, C16:0		1973	38.7 ± 0.03
3	linoleic acid, C18:2*n*6		2130	5.9 ± 0.03
4	linolenic acid, C18:3*n*3		2143	48.2 ± 0.12
5	stearic acid, C18:0		2178	4.3 ± 0.03
	SUM			100.0
	Saturated FAs			43.0
	Unsaturated FAs			57.0

^1^ The components are reported according to their elution order on apolar column (VF-1ms); ^2^ Linear Retention Indices measured on apolar column; ^3^ Linear Retention indices from literature.

**Table 4:** Chemical composition (percentage values) of dried leaves methanolic extract after derivatization, as determined by GC–MS.

N°	COMPONENTS	(%)
**Sugars**		
1	lyxose	tr
2	galactofuranose	tr
3	xylose	0.3 ± 0.02
4	sorbofuranose	0.6 ± 0.02
5	tagatofuranose	2.6 ± 0.04
6	glucose	1.0 ± 0.03
7	fructose	0.2 ± 0.01
8	glucopyranose	1.9 ± 0.02
9	allofuranose	1.7 ± 0.02
10	talofuranose	1.8 ± 0.02
11	turanose	9.3 ± 0.08
12	trehalose	12.8 ± 0.15
13	sucrose	62.3 ± 1.25
**Organic acids**		
14	lactic acid	tr
15	oxalic acid	tr
16	succinic acid	tr
17	pyruvic acid	0.1 ± 0.00
18	malonic acid	tr
**Alcohols**		
19	D-pinitol	2.8 ± 0.04
20	gycerol	1.1 ± 0.03
21	ribitol	tr
22	phytol	0.1 ± 0.00
23	myo-inositol	0.3 ± 0.02
**Fatty acids**		
24	palmitic acid	0.5 ± 0.02
25	linolenic acid	0.5 ± 0.02

tr: percentage mean values <0.1%.

**Table 5:** Metabolites identified in the 600.13 MHz 1H NMR, ^1^H-^1^H TOCSY, and ^1^H-^13^C HMBC spectra of Bligh–Dyer hydroalcoholic extracts of hop leaves in phosphate buffer/D_2_O acquired at 25 °C. Asterisks (*) indicate signals selected for integration.

Compound	Assignment	1H (ppm)	Multiplicity [J(Hz)]	^13^C (ppm)
**Sugars**				
β-D-Fructofuranose	CH-3	4.12 *		
	CH-4	4.12 *		
	CH-5	3.82		
β-D-Fructopyranose	CH-3	3.81		
	CH-5	4.00		
	CH_2_-6,6′	3.70		
α-Glucose	CH-1	5.24 *		
	CH-2	3.52		
	CH-3	3.73		
	CH-4	3.40		
	CH-5	3.84		
β-Glucose	CH-1	4.65 *		
	CH-2	3.26		
	CH-3	3.49		
	CH-4	3.40		
Sucrose	CH-1 (glucose)	5.42 *		
	CH-2	3.55		
	CH-3	3.76		73.5
	CH-4	3.48		
	CH-5	3.84		
	CH-1 (fructose)			62.3
	C-2			104.6
	CH-3	4.22	d (8.8)	
	CH-4	4.05		75.0
	CH-5	3.90		82.4
	CH2-6,6′	3.81		
**Organic acids**				
Citric acid	α,γ-CH	2.57 *	d (16.7)	45.1
	α′,γ′-CH	2.72	d (16.8)	45.1
	β-C			76.7
	COOH-1			179.4
	COOH-6			182.1
Formic acid	HCOOH	8.46 *	s	
Fumaric acid	α,β-CH=CH	6.53 *	s	
Malic acid	α-CH	4.32 *	dd (9.5; 3.1)	71.4
	β-CH	2.69	dd (15.5; 3.1)	43.7
	β′-CH	2.41	dd (15.7; 9.6)	43.7
	COOH-1			181.2
	COOH-4			180.6
Succinic acid	α, β-CH_2_	2.41 *	s	35.2
	COOH-1,4			183.3
Acetic acid	α-CH_3_	1.92 *	s	
	COOH			182.5
**Amino acids**				
Alanine	α-CH	3.78		51.5
	β-CH_3_	1.49 *,f	d (7.2)	
	COOH			176.7
GABA	α-CH_2_	2.30 *	t (7.4)	35.4
	β-CH_2_	1.91	q	24.6
	γ-CH_2_	3.02	t (7.6)	40.3
	COOH			182.5
Glutamine	α-CH	3.75		
	β,β′-CH_2_	2.15	m	
	γ-CH2	2.46	m	
Isoleucine	α-CH			60.7
	β-CH	1.99		36.9
	γ,γ′-CH_2_	1.26, 1.47		25.5
	γ-CH_3_	1.02	d (7.1)	
	δ-CH_3_	0.95 *	t (7.4)	
Leucine	β-CH_2_			40.8
	γ-CH	1.71		25.2
	δ-CH_3_	0.97 *	d (6.4)	22.9
	δ′-CH_3_	0.96 *	d (6.0)	22.5
Valine	α-CH	3.61		61.3
	β-CH	2.28		30.1
	γ-CH_3_	1.00 *	d (7.1)	17.7
	γ′-CH_3_	1.05	d (6.9)	19.0
Threonine	α-CH	3.59		61.4
	β-CH	4.25		66.9
	γ-CH_3_	1.34 *	d (6.5)	
Asparagine	α-CH	4.02		52.3
	β,β′-CH_2_	2.87 *; 2.96	dd (7.8; 17.0); dd (4.3; 17.0)	
	COOH			175.2
Aspartate	β,β′-CH_2_	2.68; 2.82 *	dd (8.8; 17.6); dd (3.8; 17.6)	
Glutamate	α-CH	3.75		
	β,β′-CH_2_	2.06; 2.14 *	m	
	γ-CH_2_	2.36	m	
Proline	α-CH	4.17		
	β,β′-CH_2_	2.08; 2.35	m, m	
	γ-CH_2_	2.02 *	m	
	δ,δ′-CH_2_	3.43; 3.34		
**Miscellaneous**				
Choline	+N(CH_3_)_3_	3.21 *	s	54.9
	α-CH_2_			68.4
Trigonelline	CH-1	9.13 *	s	
	CH_3_	4.44	s	

**Table 6:** Metabolites identified in the 600.13 MHz ^1^H NMR and ^1^H-^13^C HSQC spectra of methanolic extracts of hop leaves in methanol-*d*4 acquired at 25 °C. Asterisks (*) indicate signals selected for integration.

Compound	Assignment	^1^H (ppm)	Multiplicity [J(Hz)]	^13^C (ppm)
Polyphenols				
Xanthohumol	CH_2_-1″	3.21		
	OCH_3_	3.90	s	56.0
	CH-5′	6.02	s	
	CH-3,5	6.84	(d, 8.6 Hz)	
	CH-2,6	7.49 *	(d, 8.6 Hz)	130.6
	CH-α	7.79	(d, 15.5 Hz)	

## Data Availability

All generated data are included in this article.

## Supplementary Materials

The following supporting information can be downloaded at: https://www.mdpi.com/article/10.3390/ijms241813732/s1.
