# Supplementary material for: Metabolomic Approach Based on Analytical Techniques for the Detection of Secondary Metabolites from Humulus lupulus L. Dried Leaves
Source: Int J Mol Sci. 2023 Sep 6;24(18):13732. doi: 10.3390/ijms241813732 (PMC10531422; doi:10.3390/ijms241813732)
Supplement: Supplementary file 1 [file ijms-24-13732-s001.zip › ijms-24-13732-s001.pdf]

Supplementary Material

Metabolomic approach based on analytical techniques for the detection of secondary metabolites from *Humulus lupulus* L. dried leaves

Cosimo Taiti[1], Giacomo Di Matteo[2], Mattia Spano[2], Vittorio Vinciguerra[3], Elisa Masi[1], Luisa Mannina[2], and Stefania Garzoli[2,*]

[1] Department of Agriculture, Food, Environmental and Forest, Università di Firenze, Sesto Fiorentino, 50019 Firenze, Italy; cosimo.taiti@unifi.it (C.T.); elisa.masi@unifi.it (E.M.)
[2] Department of Chemistry and Technology of Drug, Sapienza University, 00185, Rome, Italy, giacomo.dimatteo@uniroma1.it (G.D.M.); mattia.spano@uniroma1.it (M.S); luisa.mannina@uniroma1.it (L.M.)
[3] Department for Innovation in Biological Systems, Food and Forestry, University of Tuscia, 01100, Viterbo, Italy, vincigue@unitus.it (V.V.)
* Correspondence: stefania.garzoli@uniroma1.it (S.G.)

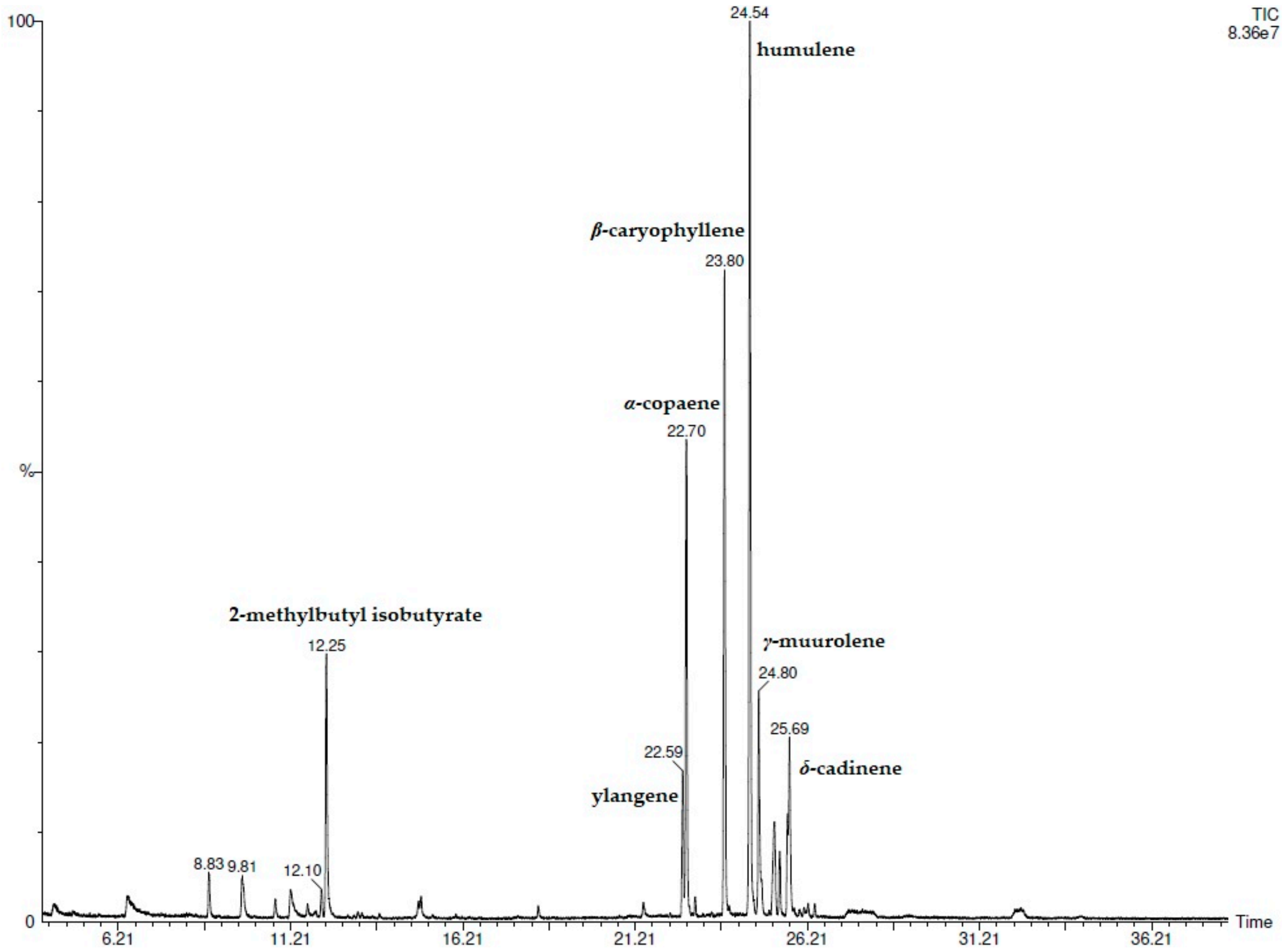

Figure S1: SPME-GC-MS chromatogram of the dried hop leaves

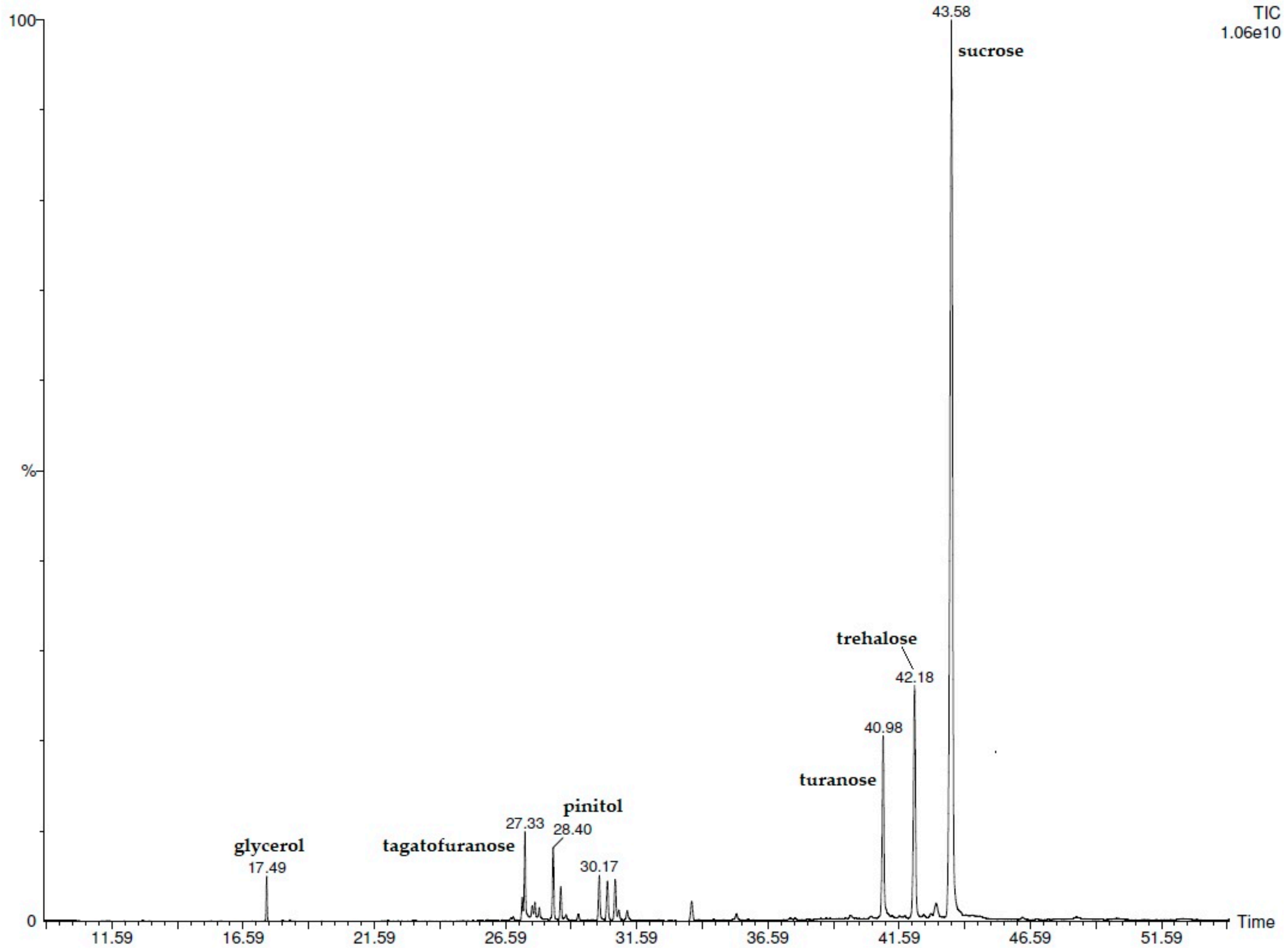

Figure S2: GC-MS chromatogram of the methanolic extract after derivatization.

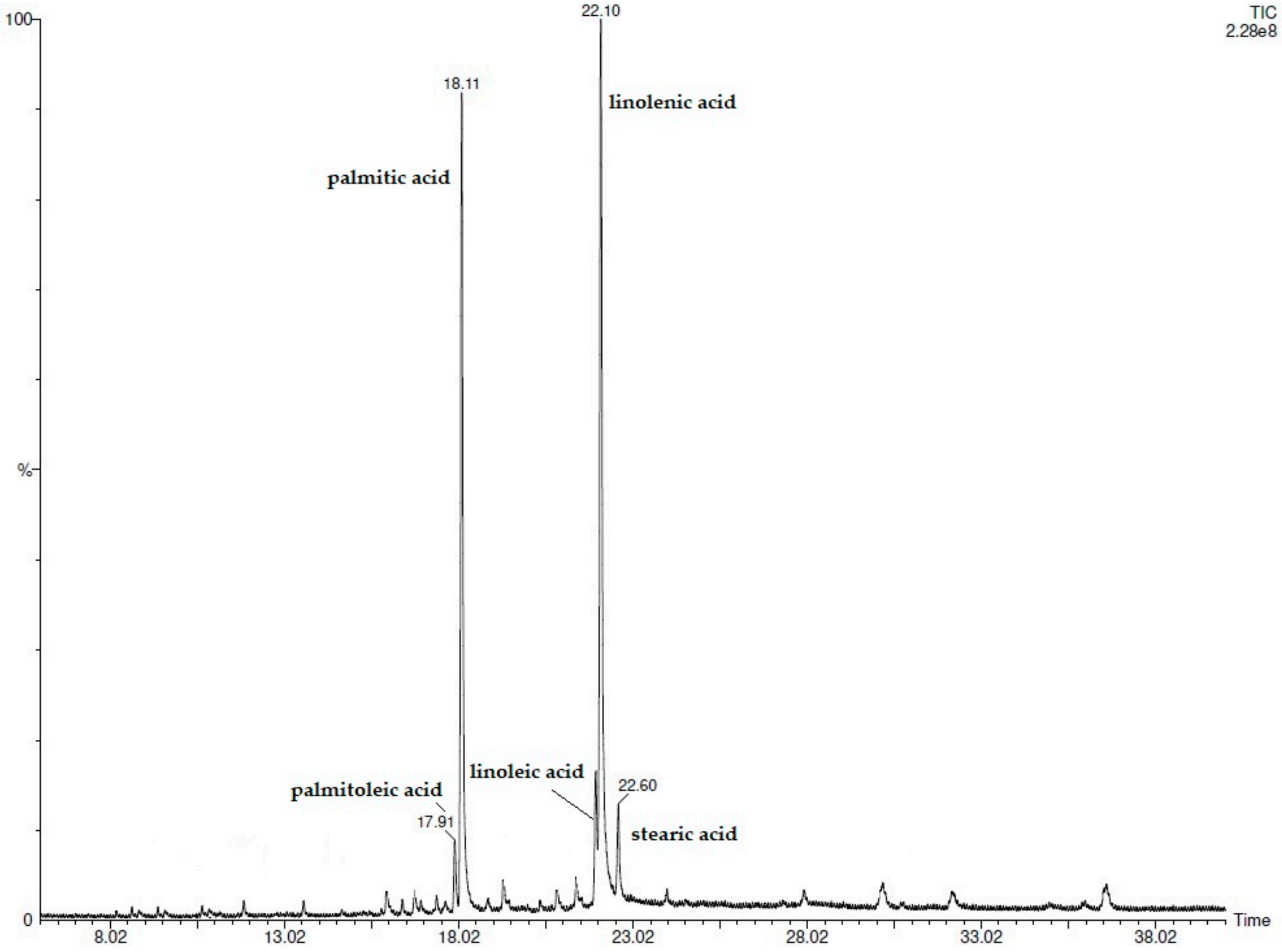

Figure S3: GC-MS chromatogram of the transesterified dried leaves extract.

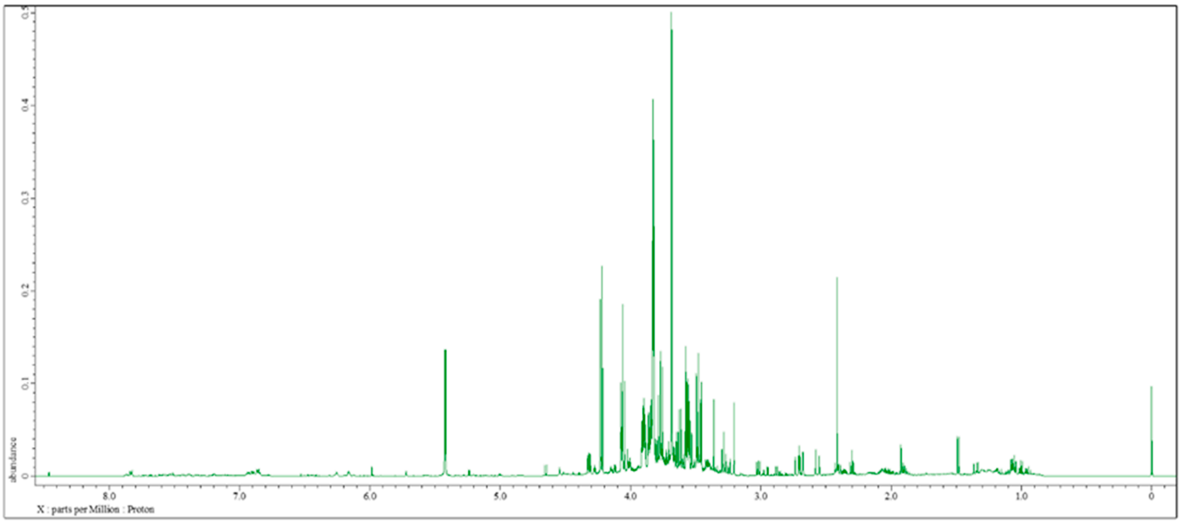

Figure S4: ^{1}H NMR spectrum of the hydroalcoholic hop leaves fraction in phosphate buffer/D_2O.

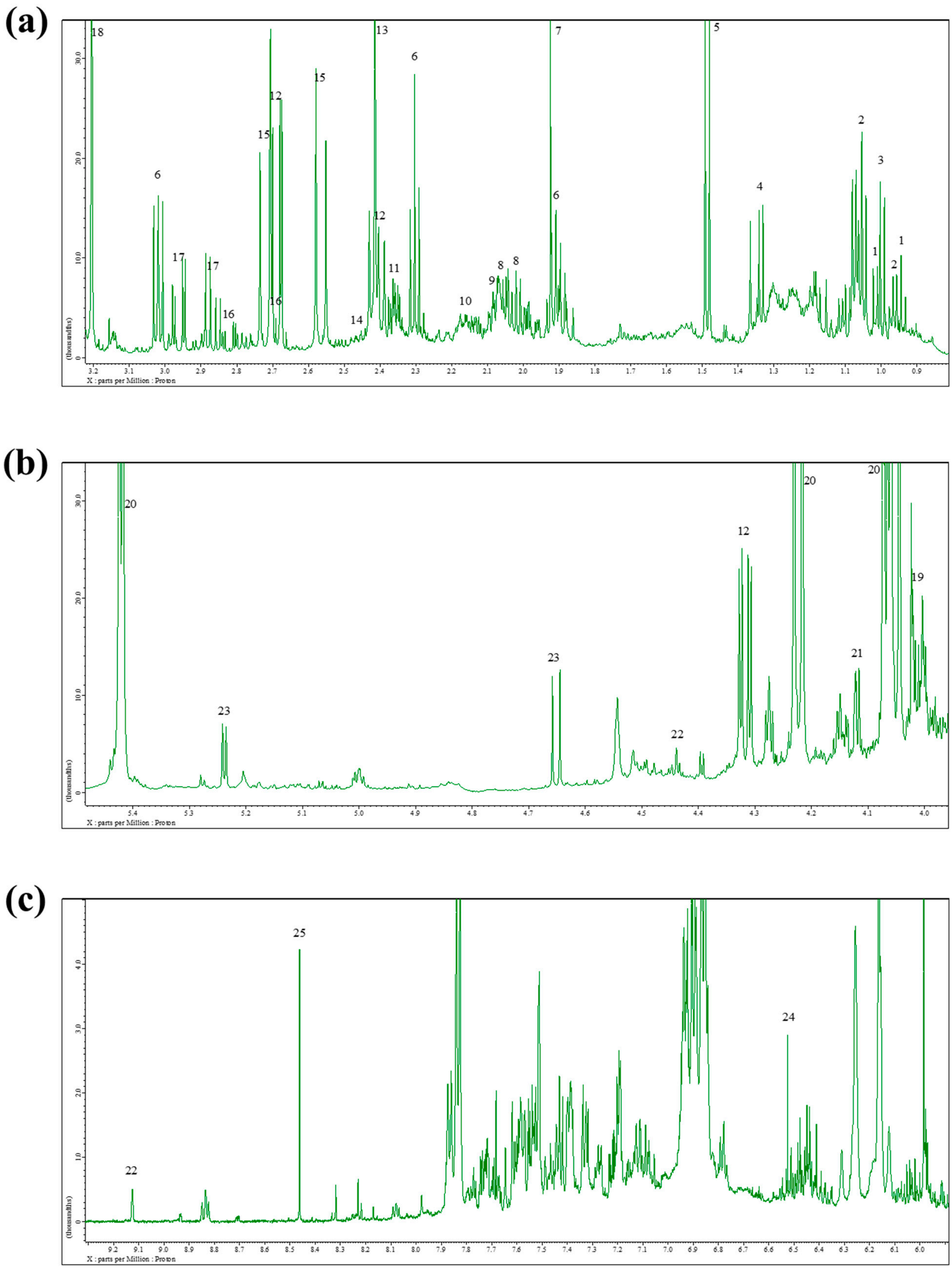

Figure S5. Expansions of ^{1}H NMR spectrum of the hydroalcoholic hop leaves fraction in phosphate buffer/D_2O: (a) Upfield region; (b) Middle field region; (c) Downfield region.

Assignments: 1, Isoleucine; 2, Leucine; 3, Valine; 4, Threonine; 5, Alanine; 6, GABA; 7, Acetate; 8, Proline; 9, Glutamate; 10, Glutamate + Glutamine; 11, Glutamate + Proline; 12, Malic acid; 13, Succinate; 14, Glutamine; 15, Citrate; 16, Aspartate; 17, Asparagine; 18, Choline; 19, β-D-Fructopyranose; 20, Sucrose; 21, β-D-Fructofuranose; 22, Trigonelline; 23, Glucose; 24, Fumarate; 25, Formate.

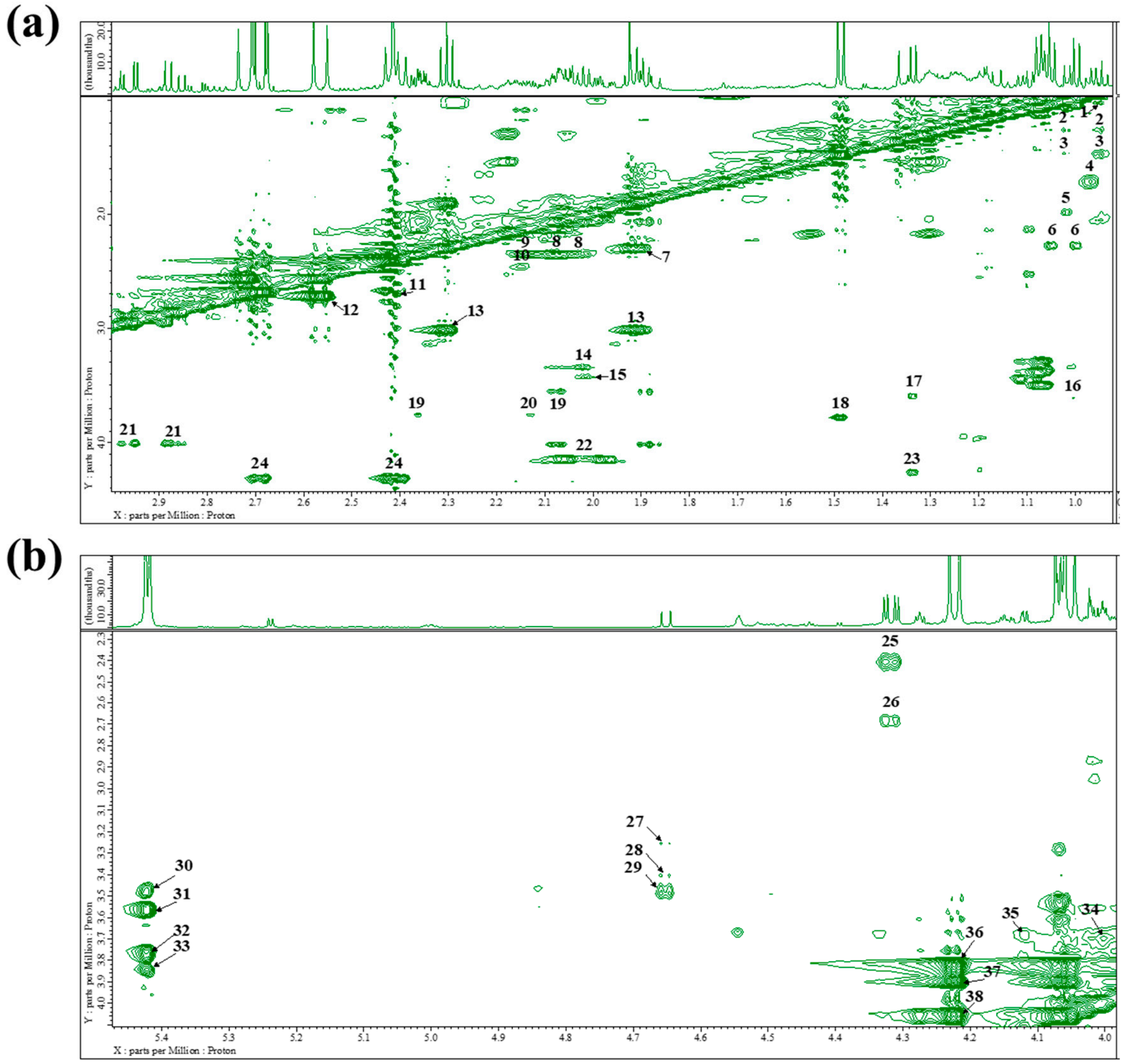

Figure S6. Expansions of TOCSY spectrum of hydroalcoholic fraction of hop leaves in phosphate buffer/D_2O: (a) Upfield region; (b) Middle field region.

Key: Iso, Isoleucine; Leu, Leucine; Val, Valine; Thr, Threonine; Ala, Alanine; Glu, Glutamate; Gln, Glutamine; Pro, Proline; MalA, Malate; CitA, Citrate; Asp, Aspartate; β-Glu, β-Glucose; Suc, Sucrose; β-Fru-pyr, β-D-Fructopyranose; β-Fru-fur, β-Fructofuranose;

Assignments: 1, γ-CH_3 Iso; 2, γ'-CH_2 Iso; 3, γ-CH_2 Iso; 4, γ-CH Leu; 5, β-CH Iso; 6, β-CH Val; 7, α-CH_2 GABA; 8, β-CH_2 Pro; 9, γ-CH_2 Glu; 10, γ-CH_2 Gln; 11, β-CH MalA; 12, α',γ'-CH CitA; 13, γ-CH_2 GABA; 14, δ-CH_2 Pro; 15, δ′-CH_2 Pro; 16, α-CH Val; 17, β-CH Thr; 18, α-CH Ala; 19, α-CH Glu; 20, α-CH Glu, α-CH Gln; 21, α-CH Asp; 22, α-CH Pro; 23, β-CH Thr; 24, α-CH MalA; 25, β′-CH MalA; 26, β-CH MalA; 27, CH2 β-Glu; 28, CH4 β-Glu; 29, CH3 β-Glu; 30, CH4 Suc;

31, CH2 Suc; 32, CH3 Suc; 33, CH5 Suc; 34, CH6 β-Fru-pyr; 35, CH6 β-Fru-fur; 36, CH6′ Suc; 37, CH5′ Suc; 38, CH4′ Suc.

(a)

(b)

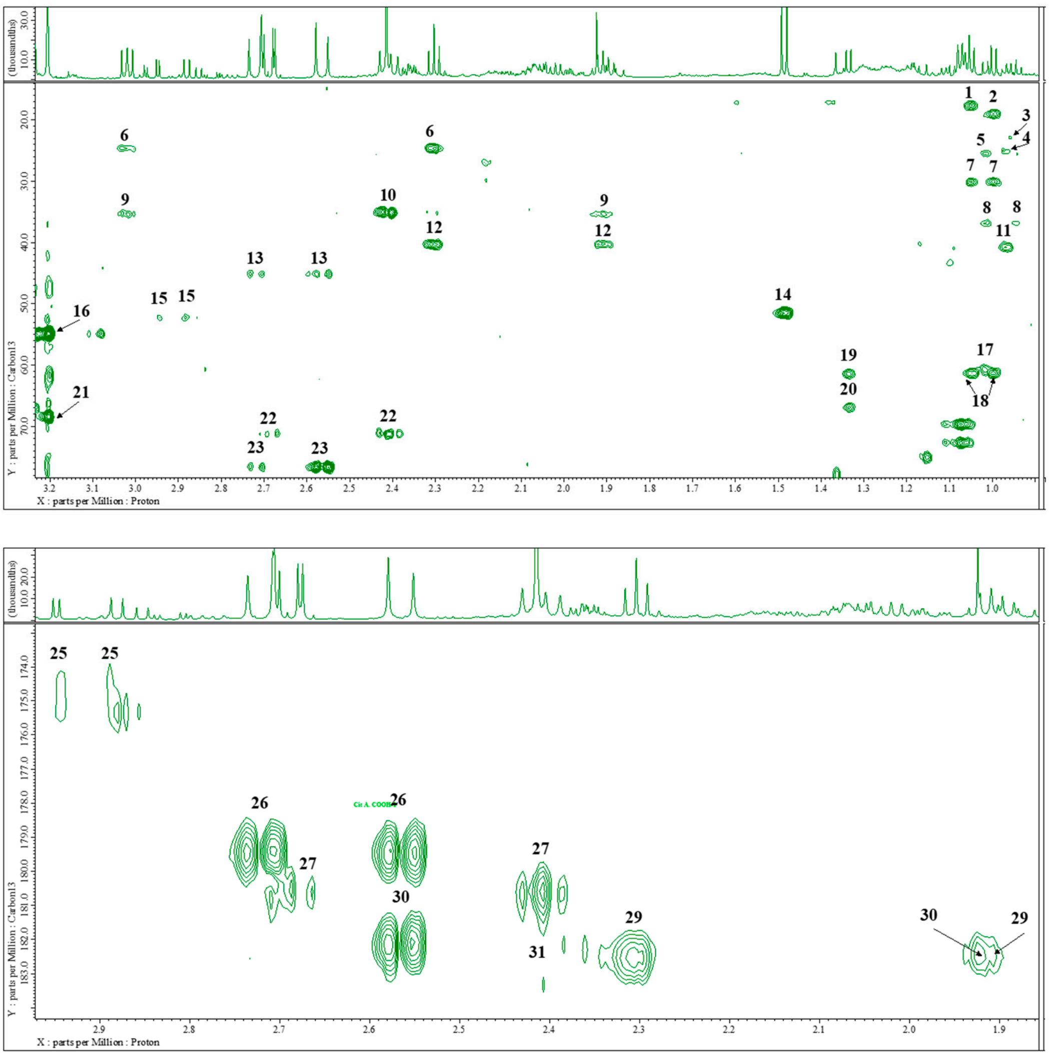

Figure S7. Expansions of HMBC spectrum of the hydroalcoholic hop leaves fraction in phosphate buffer/D_2O: (a) Upfield region; (b) Carboxylic region.

Key: Iso, Isoleucine; Leu, Leucine; Val, Valine; Thr, Threonine; Ala, Alanine; Glu, Glutamate; Gln, Glutamine; Pro, Proline; MalA, Malate; CitA, Citrate; Asp, Aspartate; β-Glu, β-Glucose; Suc, Sucrose; β-Fru-pyr, β-D-Fructopyranose; β-Fru-fur, β-Fructofuranose; SucA, Succinate; Asn, Asparagine; Cho, Choline;

Assignments: 1, γ-CH_3 Val; 2, γ′-CH_3 Val; 3, δ-CH_3 Leu; 4, γ-CH Leu; 5, γ-CH_2 Iso; 6, β-CH_2 GABA; 7, β-CH Val; 8, β-CH Iso; 9, α-CH GABA; 10, α,β-CH_2 SucA; 11, β-CH_2 Leu; 12, γ-CH_2 GABA; 13, CH CitA; 14, α-CH Ala; 15, α-CH Asn; 16, N(CH3)3 Cho; 17, α-CH Iso; 18, α-CH Val; 19, α-CH Thr; 20, β-CH Thr; 21, α-CH_2 Cho; 22, α-CH MalA; 23, β-C CitA; 24, COOH Ala; 25, COOH Asn; 26, COOH-1 CitA; 27, COOH-4 MalA; 28, COOH GABA; 29, COOH AceA; 30, COOH-6 CitA; 31, COOH 1,4 SucA.

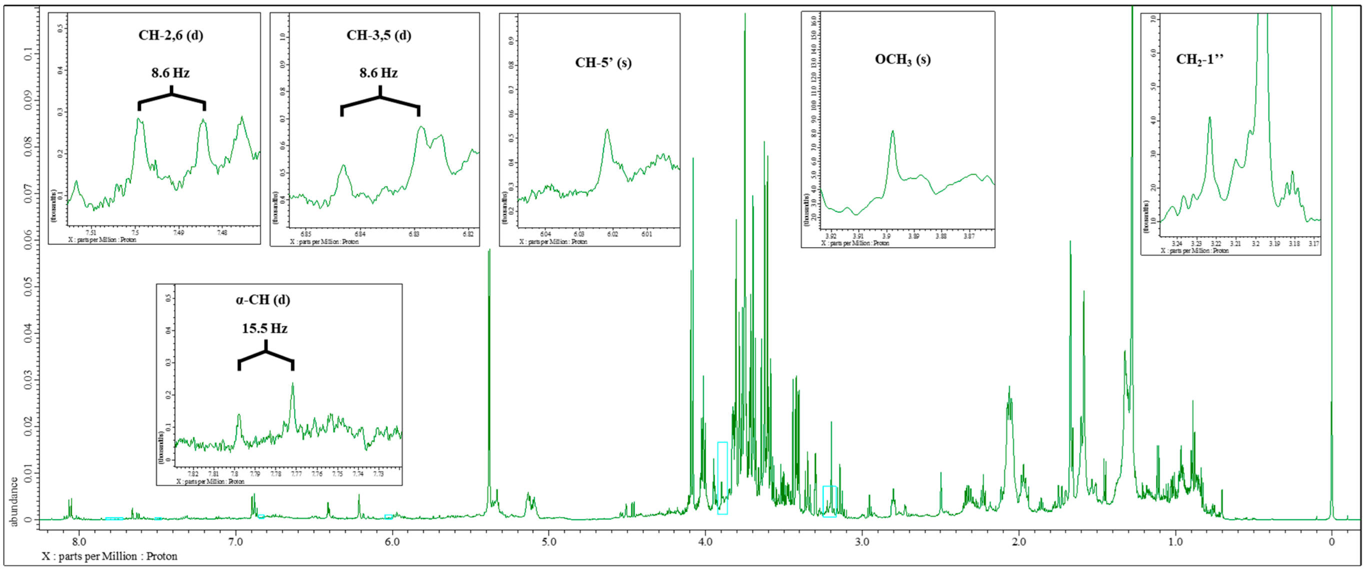

Figure S8. ^{1}H NMR spectrum of the methanolic hop leaves extract in methanol-d4 with the expansion of xanthohumol assignments.

NMR quantification

For the absolute quantification of each target compound the following equation was applied to convert the integrated peak area to a concentration in mg/100g:

$$c_T = \frac{I_T}{I_{St}} \times \frac{x_{St}}{x_T} \times \frac{c_{St}}{m_M} \times \frac{V_{St}}{10} \times M_T$$

C_T concentration of the target compound in the hop leaves matrix [mg/100g];

M_T molecular weight of target compound [g/mol]

I_T relative integral value of ^{1}H NMR signal of the target compound

I_{St} relative integral value of ^{1}H NMR signal of the standard compound

x_{St} number of protons belonging to the ^{1}H NMR signal of the standard compound (9 for TSP internal standard)

x_T number of protons belonging to the ^{1}H NMR signal of the target compound

c_{St} concentration of standard compound in the solution used for ^{1}H NMR measurement [mmol/L]

v_{St} volume of solution used for ^{1}H NMR measurement [mL]

m_M weight of hop leaves matrix used for the extraction [g]

10 factor to convert the final concentration to mg/100g

Table S1. Chemical volatile composition (with identifiers: InChlkey) of the dried hop leaves as determined by SPME-GC-MS.

N°	COMPONENT[1]	Identifiers
1	isobutyric acid	KQNPFQTWMSNSAP-UHFFFAOYSA-N
2	propionic acid	XBDQKXXYIPTUBI-UHFFFAOYSA-N
3	5-hepten-2-one, 6-methyl-	UHEPJGULSIKKTP-UHFFFAOYSA-N
4	2-methylbutyl isobutyrate	DUAXUBMIVRZGCO-UHFFFAOYSA-N
5	*β*-myrcene	UAHWPYUMFXYFJY-UHFFFAOYSA-N
6	amyl isovalerate	QURFFFCYNQXLCU-UHFFFAOYSA-N
7	*β*-cyclocitral	MOQGCGNUWBPGTQ-UHFFFAOYSA-N
8	ylangene	VLXDPFLIRFYIME-XTLGRWLVSA-N
9	*α*-copaene	VLXDPFLIRFYIME-UHFFFAOYSA-N
10	*β*-bourbonene	YIRAHEODBQONHI-XTLGRWLVSA-N
11	*β*-caryophyllene	NPNUFJAVOOONJE-IOMPXFEGSA-N
12	*α*-humulene	FAMPSKZZVDUYOS-HRGUGZIWSA-N
14	*γ*-muurolene	WRHGORWNJGOVQY-ZNMIVQPWSA-N
13	*β*-eudesmene	YOVSPTNQHMDJAG-ZNMIVQPWSA-N
15	*α*-selinene	OZQAPQSEYFAMCY-GIJJTGMTSA-N
16	*γ*-cadinene	WRHGORWNJGOVQY-RBSFLKMASA-N
17	*δ*-cadinene	FUCYIEXQVQJBKY-HIFRSBDPSA-N
18	selina-3,7(11)-diene	WNRBYZQFEBIUGD-CABCVRRESA-N

Table S2. Chemical composition (with identifiers: InChlkey) of *H. lupulus* dried leaves identified via PTR-ToF-MS.

N° of compounds	*m/z*	Chemical Formula	Tentative Identification	Identifiers
1	27.022	$C_2H_3^+$	Acetylene	HSFWRNGVRCDJHI-UHFFFAOYSA-N
2	31.018	CH_3O^+	Formaldehyde	WSFSSNUMVMOOMR-UHFFFAOYSA-N
3	33.033	CH_5O^+	Methanol	OKKJLVBELUTLKV-UHFFFAOYSA-N
4	41.038	$C_3H_5^+$	Alkylic fragment	not determinable
5	43.018	$C_2H_3O^+$	Aldehyde fragment	not determinable
6	43.054	$C_3H_7^+$	General alkane/VOC fragment	not determinable
7	45.033	$C_2H_5O^+$	Acetaldehyde	IKHGUXGNUITLKF-UHFFFAOYSA-N
8	47.013	$CH_3O_2^+$	Formic acid/formates	not determinable
9	49.011	CH_5S^+	S Compound (methanethiol)	not determinable
10	55.054	$C_4H_7^+$	Fragment	not determinable
11	57.069	$C_4H_9^+$	Alcohol fragment	not determinable
12	59.049	$C_3H_7O^+$	Propanal, Acetone	not determinable
13	61.028	$C_2H_5O_2^+$	Acetates	not determinable
14	69.069	$C_5H_9^+$	Isoprene/Cycloalkane fragment	not determinable
15	71.049	$C_4H_7O^+$	Butenal	not determinable
16	73.065	$C_4H_9O^+$	Isobutanal/butanone/methylpropanal	not determinable
17	83.086	$C_6H_{11}^+$	C6 compounds (hexenal, hexenols)	not determinable
18	85.065	$C_5H_9O^+$	Methyl butenal	not determinable

19	87.044	$C_4H_7O_2^+$	2,3-Butanedione; Butyrolactone	not determinable
20	87.080	$C_5H_{11}O^+$	Pentanal/3-methylbutanal	not determinable
21	93.069	$C_7H_9^+$	Terpene fragment	not determinable
22	107.086	$C_8H_{11}^+$	Terpene fragment	not determinable
23	109.101	$C_8H_{13}^+$	Terpene fragment	not determinable
24	133.101	$C_{10}H_{13}^+$	Terpene fragment	not determinable
25	205.195	$C_{15}H_{25}$+	Sesquiterpenes like compounds	not determinable

Table S3. FAs content (with identifiers: InChlKey) of the transesterified extract, as determined by GC-MS.

N°	COMPONENT[1]	Identifiers
1	palmitoleic acid, C16:1*n*7	SECPZKHBENQXJG-FPLPWBNLSA-N
2	palmitic acid, C16:0	IPCSVZSSVZVIGE-UHFFFAOYSA-N
3	linoleic acid, C18:2*n*6	OYHQOLUKZRVURQ-HZJYTTRNSA-N
4	linolenic acid, C18:3*n*3	DTOSIQBPPRVQHS-PDBXOOCHSA-N
5	stearic acid, C18:0	QIQXTHQIDYTFRH-UHFFFAOYSA-N

Table S4. Chemical composition (with identifiers: InChlKey) of dried leaves methanolic extract after derivatization, as determined by GC-MS.

N°	COMPONENTS	(%)
	**Sugars**	
1	D-lyxose	SRBFZHDQGSBBOR-AGQMPKSLSA-N
2	galactofuranose	AVVWPBAENSWJCB-RSVSWTKNSA-N
3	xylose	PYMYPHUHKUWMLA-VPENINKCSA-N
4	sorbofuranose	RFSUNEUAIZKAJO-IANNHFEVSA-N
5	tagatofuranose	RFSUNEUAIZKAJO-OEXCPVAWSA-N
6	glucose	WQZGKKKJIJFFOK-MDMQIMBFSA-N
7	fructose	BJHIKXHVCXFQLS-UYFOZJQFSA-N
8	D-glucopyranose	WQZGKKKJIJFFOK-GASJEMHNSA-N
9	allofuranose	AVVWPBAENSWJCB-CBPJZXOFSA-N
10	talofuranose	AVVWPBAENSWJCB-QTVWNMPRSA-N
11	turanose	RULSWEULPANCDV-PIXUTMIVSA-N
12	trehalose	HDTRYLNUVZCQOY-LIZSDCNHBN
13	sucrose	CZMRCDWAGMRECN-SFOFJGFUSA-N
	**Organic acids**	
14	lactic acid	JVTAAEKCZFNVCJ-UHFFFAOYSA-N
15	oxalic acid	MUBZPKHOEPUJKR-UHFFFAOYSA-N
16	succinic acid	KDYFGRWQOYBRFD-UHFFFAOYSA-N
17	pyruvic acid	LCTONWCANYUPML-UHFFFAOYSA-N
18	malonic acid	OFOBLEOULBTSOW-UHFFFAOYSA-N
	**Alcohols**	

19	D-pinitol	DSCFFEYYQKSRSV-FEPQRWDDSA-N
20	gycerol	PEDCQBHIVMGVHV-UHFFFAOYSA-N
21	ribitol	HEBKCHPVOIAQTA-NGQZWQHPSA-N
22	phytol	BOTWFXYSPFMFNR-PYDDKJGSSA-N
23	myo-inositol	CDAISMWEOUEBRE-UHFFFAOYSA-N

Table S5. Metabolites identified in the 600.13 MHz 1H NMR, ^{1}H- ^{1}H TOCSY and ^{1}H-^{13}C HMBC spectra of Bligh–Dyer hydroalcoholic extracts (with identifiers: InChlKey) of hop leaves in phosphate buffer/D_2O acquired at 25 °C.

Compound	Identifiers
β-D-Fructofuranose	RFSUNEUAIZKAJO-ARQDHWQXSA-N
β-D-Fructopyranose	LKDRXBCSQODPBY-ARQDHWQXSA-N
α-Glucose	WQZGKKKJIJFFOK-MDMQIMBFSA-N
β-D-Glucose	WQZGKKKJIJFFOK-VFUOTHLCSA-N
Sucrose	CZMRCDWAGMRECN-SFOFJGFUSA-N
Citric acid	KRKNYBCHXYNGOX-UHFFFAOYSA-N
Formic acid	BDAGIHXWWSANSR-UHFFFAOYSA-N
Fumaric acid	VZCYOOQTPOCHFL-OWOJBTEDSA-N
Malic acid	BJEPYKJPYRNKOW-UHFFFAOYSA-N
Succinic acid	KDYFGRWQOYBRFD-UHFFFAOYSA-N
Acetic acid	QTBSBXVTEAMEQO-UHFFFAOYSA-N
Alanine	QNAYBMKLOCPYGJ-UWTATZPHSA-N
GABA	BTCSSZJGUNDROE-UHFFFAOYSA-N
Glutamine	ZDXPYRJPNDTMRX-GSVOUGTGSA-N
Isoleucine	AGPKZVBTJJNPAG-CRCLSJGQSA-N
Leucine	ROHFNLRQFUQHCH-RXMQYKEDSA-N
Valine	KZSNJWFQEVHDMF-SCSAIBSYSA-N
Threonine	AYFVYJQAPQTCCC-STHAYSLISA-N
Asparagine	DCXYFEDJOCDNAF-UWTATZPHSA-N
Aspartate	CKLJMWTZIZZHCS-REOHCLBHSA-N
Glutamate	WHUUTDBJXJRKMK-UHFFFAOYSA-M
Proline	ONIBWKKTOPOVIA-SCSAIBSYSA-N
Choline	OEYIOHPDSNJKLS-UHFFFAOYSA-N
Trigonelline	WWNNZCOKKKDOPX-UHFFFAOYSA-N
